# Recent Advances in Investigating Functional Dynamics of Chromatin

**DOI:** 10.3389/fgene.2022.870640

**Published:** 2022-04-05

**Authors:** Xiangyan Shi, Ziwei Zhai, Yinglu Chen, Jindi Li, Lars Nordenskiöld

**Affiliations:** ^1^ Department of Biology, Shenzhen MSU-BIT University, Shenzhen, China; ^2^ School of Biological Sciences, Nanyang Technological University, Singapore, Singapore

**Keywords:** NMR, FRET, MD simulations, dynamics of nucleosomes, nucleosome conformational dynamics

## Abstract

Dynamics spanning the picosecond-minute time domain and the atomic-subcellular spatial window have been observed for chromatin *in vitro* and *in vivo*. The condensed organization of chromatin in eukaryotic cells prevents regulatory factors from accessing genomic DNA, which requires dynamic stabilization and destabilization of structure to initiate downstream DNA activities. Those processes are achieved through altering conformational and dynamic properties of nucleosomes and nucleosome–protein complexes, of which delineating the atomistic pictures is essential to understand the mechanisms of chromatin regulation. In this review, we summarize recent progress in determining chromatin dynamics and their modulations by a number of factors including post-translational modifications (PTMs), incorporation of histone variants, and binding of effector proteins. We focus on experimental observations obtained using high-resolution techniques, primarily including nuclear magnetic resonance (NMR) spectroscopy, Förster (or fluorescence) resonance energy transfer (FRET) microscopy, and molecular dynamics (MD) simulations, and discuss the elucidated dynamics in the context of functional response and relevance.

## Introduction

**FIGURE 1:**
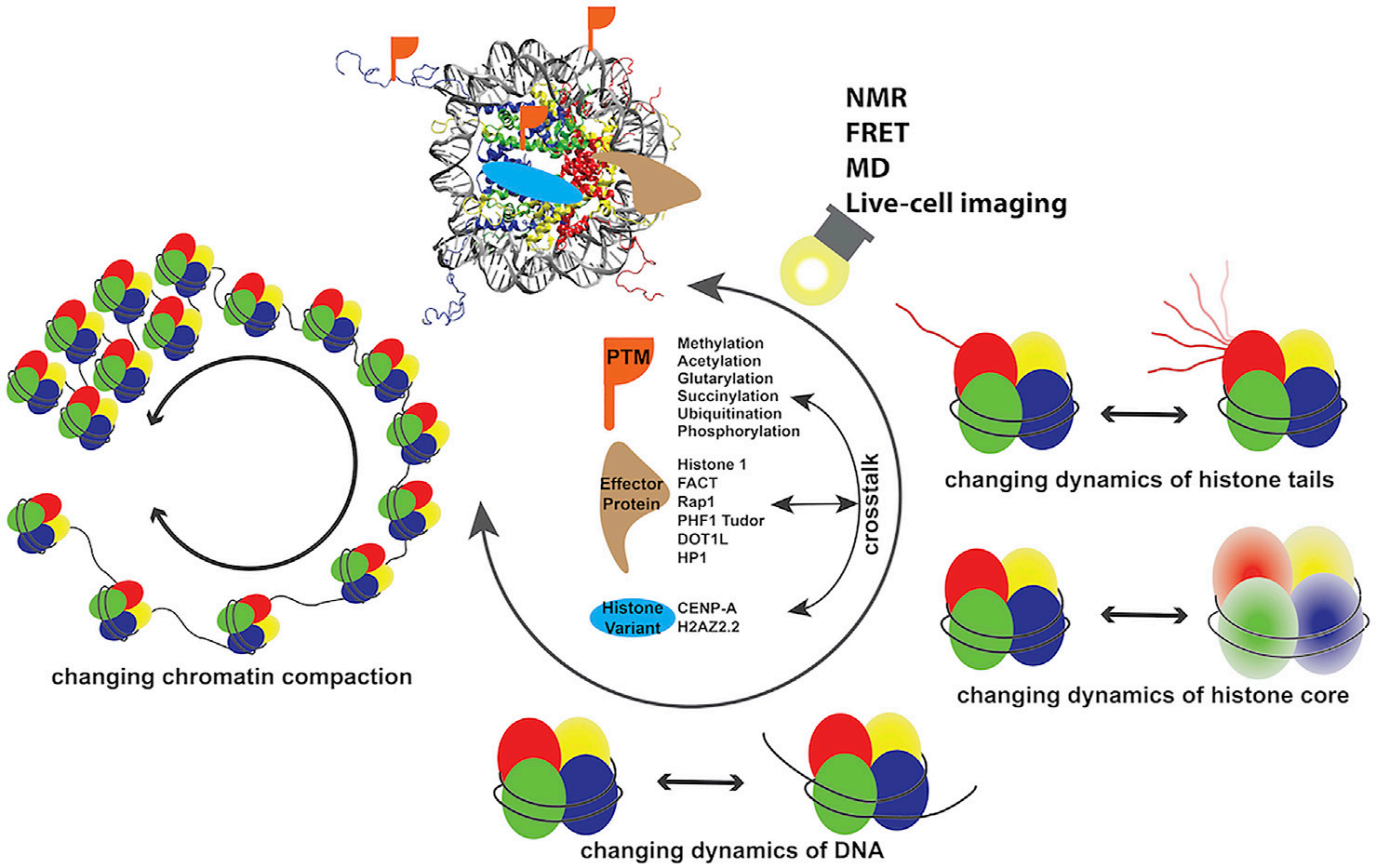
Dynamics of chromatin modulated by a number of factors discussed in this review.

Chromatin in eukaryotic cells is organized in the form of 147 bp DNA wrapping the histone octamer (HO) complex to form nucleosome core particles (NCPs), connected by linker DNA to form a “beads-on-a-string,” which in the presence of linker histone and/or physiological salt, condenses to higher ordered structures (Zhou et al., 2019; Baldi et al., 2020). This condensed structure acts as the barrier for protein factors necessary for accessing DNA during downstream genomic activities and requires dynamic stabilization and destabilization for maintaining cellular homeostasis. The accomplishment of genomic DNA activities in eukaryotic cells is propagated from the modulation of dynamic spatiotemporal organization of chromatin, which is achieved through factors including post-translational modifications (PTMs) (Jenuwein and Allis, 2001; Bannister and Kouzarides, 2011; Bowman and Poirier, 2014; Fenley et al., 2018), incorporation histone variants (Talbert and Henikoff, 2016; Martire and Banaszynski, 2020), remodelers, and other effector proteins (Tyagi et al., 2016; Armeev et al., 2019; Reyes et al., 2021). Since the first atomic resolution structure was obtained 24 years ago (Luger et al., 1997), well over a hundred structures of NCPs with different DNA sequences or histone variants and in complex with protein factors have been determined by X-ray diffraction (XRD) and cryogenic electron microscopy (cryo-EM) (Luger et al., 1997; Korolev et al., 2018; Zhou et al., 2019; Soman et al., 2020; Lobbia et al., 2021). The atomic structure information opened the door to understanding the molecular basis of genomic DNA regulation processes. Various NCPs adopt structures with high similarity and minor local conformational differences, suggesting that molecular characteristics beyond structure also play dominant roles in the biological behaviors of chromatin associated with incorporation of different histone variants, modifications, and DNA sequences. Recent studies have determined the dynamics properties of several nucleosomes and nucleosome–protein complexes, revealing the link between biological function and dynamics properties. Dynamics of chromatin span from picosecond to minute timescales at atomic to subcellular levels, which greatly contribute to regulating various DNA processes and remain largely unclear at high spatiotemporal resolution. With the recent development of high-resolution techniques primarily including nuclear magnetic resonance (NMR) spectroscopy, Förster (or fluorescence) resonance energy transfer (FRET) microscopy, and molecular dynamics (MD) simulations, increasing information on dynamics of nucleosomes and nucleosome–protein complexes have been determined, suggesting the functional components of this important molecular property. In this review, we focus on recent research investigating the dynamics of chromatin systems (Figure 1) and we discuss the biological roles of these functional dynamics features.

### Advanced Techniques for Characterizing Chromatin Dynamics

Recent development of advanced techniques primarily including NMR, FRET, and MD simulations has significantly stimulated *in vitro* research on chromatin dynamics. NMR allows for quantifying the motional amplitudes and timescales for dynamics covering second-picosecond timescales at atomic resolution (Krushelnitsky et al., 2013; Kovermann et al., 2016; Shi and Rienstra, 2016). Solution-state NMR has been successfully implemented to determine the conformation and dynamics of nucleosomes. It mainly provides information of the highly flexible histone tails (Zhou et al., 2012; Morrison et al., 2018; Ohtomo et al., 2021; Rabdano et al., 2021) or methyl sites in the rigid histone core (Kato et al., 2011; Kitevski-LeBlanc et al., 2018) because of its limitation in detecting rigid structural components of large molecules. This intrinsic size limitation is overcome by using solid-state NMR (SSNMR) that has developed as an emerging powerful technique in studying chromatin. This revealed structure and dynamics for several nucleosomes and nucleosome–protein complexes (Ackermann and Debelouchina, 2021; le Paige et al., 2021). NMR techniques require isotope labeling to gain sufficient sensitivity and sometimes also require fragment labeling (e.g., labeling one of the histones) to reduce signal complexity. Preparation of large amounts (milligrams) of homogenous nucleosome complexes with isotope labeling for NMR characterization is not always trivial and demands plenty of effort. FRET, particularly single-molecule FRET (smFRET), offers a highly sensitive and suitable approach to probe the conformational dynamics of chromatin (Buning and van Noort, 2010; Sasmal et al., 2016; Kilic et al., 2018). Typically, the fluorophore pairs are installed at specific sites of the DNA in nucleosomes and their distances between 1 and 10 nm can be derived from the FRET efficiency. The experimental data reflect the transitions of distinguished states originating from dynamics such as DNA wrapping/unwrapping in nucleosomes (Kilic et al., 2018). Site-specific labeling at particular sites with suitable fluorophores is generally a challenging task for nucleosomes and nucleosome–protein complexes. The spatial resolution limit of FRET prevents its access to local structural details at the atomic resolution (Sasmal et al., 2016). For this reason, it is often integrated with other techniques such as NMR and/or MD to delineate the atomistic pictures of conformations. Another superior technique, MD simulation, permits investigating structure and multi-scale dynamics at the atomic level for chromatin (Huertas and Cojocaru, 2021). All-atom MD simulations of mononucleosomes have reached a timescale of up to 15 ms (Armeev et al., 2021; Huertas and Cojocaru, 2021) and can detect key atomistic characteristics that modulate the dynamics of nucleosomes. Because of the limitation of all-atom MD, coarse-grained MD has been established to simulate nucleosomes at a longer timescale and capture the organization and dynamics of nucleosome arrays (Voltz et al., 2008; Huertas and Cojocaru, 2021). Future development of force fields, water models, and supercomputer systems is required to improve the accuracy of MD. This will enable extension of the simulation timescale toward milliseconds and studying longer nucleosome arrays that can capture important functionally relevant atomistic features. Despite the current technical limitations, the application of these three techniques provides substantial new insights into the dynamics of chromatin with various modulators as discussed in the following sections.

The dynamics of chromatin *in vivo* cover a wide spatiotemporal window across the entire cell cycle, which is hardly detectable in real time by conventional characterization tools. FRET-based visualization of chromatin is a powerful tool to track the dynamic states of chromatin in live cells. To date, the focus in this field has been largely placed on designing proper biosensors (Llères et al., 2009; Sasaki et al., 2009; Sanchez et al., 2017; Peng et al., 2018; Gong et al., 2021; Mendonca et al., 2021). With the recent efforts toward this direction, studies detected dynamic fluctuations in histone H4K5 and K8 acetylation in living cells and confirmed that H4K5 acetylation is significantly reduced during mitosis (Sasaki et al., 2009). Another study revealed that H3S10p attenuates H3K9me3 at the onset of mitosis during a cell cycle, and demethylation of H3K9me3 is accompanied by the reduction of heterochromatin-like structures and thereby may increase the accessibility and promote the recruitment of chromatin remodelers (Peng et al., 2018). Although the design of proper biosensors is tedious and challenging, those examples of FRET-based visualization demonstrate its advances in tracking spatial distribution and abundance of epigenetic marks at the subcellular levels, which provides indispensable information in chromatin biology research.

### Functional Dynamics of Nucleosomes

Recent molecular level NMR and MD studies covering nanosecond to millisecond timescales successfully demonstrated that in addition to structural characteristics, nucleosome dynamics provide important functional relevance. NMR studies determined conformational dynamics in NCPs for both highly flexible N-terminal tails and plastic histone core (Kitevski-LeBlanc et al., 2018; Shi et al., 2018; Xiang et al., 2018; Shi et al., 2020a; Shi et al., 2020b; Rabdano et al., 2021; Zandian et al., 2021). Histone tails in nucleosomes are the most well-characterized regions in studies of dynamics at the atomic level. Because of the highly flexible properties of these N-terminal tails, the atomistic pictures of conformations and dynamics are primarily captured by NMR and MD simulations (Massiah et al., 2013; Musselman et al., 2013; Morrison et al., 2018; Armeev et al., 2019; Abramov et al., 2020; Shi et al., 2020a; Ohtomo et al., 2021). A recent solution-state NMR study characterized the H2A and H2B tails in nucleosomes using deuterated samples at an ultra-high magnetic field (950 MHz), which observed two conformations of the tails corresponding to states interacting with different DNA regions (Ohtomo et al., 2021). It was noted that the observed stable conformations represent the averaged conformations of a large assembly of N-terminal tail states that likely involve fast exchange. Recent advances in SSNMR studies of chromatin allows elucidating the structure and dynamics for both the highly flexible tails and the rigid core for samples in compact states, where the water contents of the nucleosome samples are around 50–90% (Gao et al., 2013; Shi et al., 2018; Xiang et al., 2018; Ackermann and Debelouchina, 2021; Zandian et al., 2021). The determined motional amplitudes for amino acid backbone groups of histones in the NCPs suggest that motions at the nanosecond-microsecond timescale closely correlate with the structures (Shi et al., 2018; Shi et al., 2020a). More importantly, it revealed that there are collective microsecond-millisecond motions present at multiple regions of histones that form particular pathways to possibly transmit epigenetic signals form the NCP core to DNA sites distant from the histone sites (Shi et al., 2018; Shi et al., 2020b). Such studies of dynamics at the molecular level allow us to understand the functional dynamic properties and their contributions in DNA regulation activities. Consistent with this, a solution-state NMR study of nucleosomes harboring tetra-acetylated H4 revealed that acetylation shifts H3 tail dynamic conformations to being more dominant in the DNA–histone contact state, suggesting the existence of a histone tail network (Furukawa et al., 2020). Taken together, these studies suggest that dynamic networks likely extended from the HO to remote DNA sites. The coupling between DNA and histone conformation and dynamics on the microsecond timescale was directly observed by MD studies (Shaytan et al., 2016; Winogradoff and Aksimentiev, 2019; Armeev et al., 2021). The 15-microsecond all-atom MD simulation captured the atomistic details and illustrated that DNA breathing/unwrapping events occur at multi-microsecond timescale and are governed by histone dynamics (Armeev et al., 2021), which also demonstrated the functional roles of the plasticity of histone core in nucleosomes. Sub-nucleosomes including hexsomes and tetrasomes are species that also contribute to the regulation of DNA processes. The combination of NMR and MD studies elucidated that the H3 tails in hexasome possess distinct and asymmetric formations, and dynamics of the tails are increased with the loss of H2A/H2B dimer in nucleosome (Morrison et al., 2021). Similarly, a FRET study proposed a step-wise disassembly process and determined a shorter opening timescale for hexasomes in comparison with nucleosomes, indicating that the dissociation of a H2A/H2B dimer led to a more accessible DNA (Gansen et al., 2018). In addition to internal dynamics faster than microseconds, motions of hundreds of milliseconds were detected for nucleosome arrays (a mimic of chromatin fiber), which is the interconverting of different tetranucleosome stacking registers that can be modulated through long-range regulation factors to accomplish biological functions (Kilic et al., 2018).

### Post-Translational Modifications

PTMs are one of the most common epigenetic regulatory mechanisms in eukaryotic proteins (Jenuwein and Allis, 2001). The modifications typically occur at signal amino acid sites of histones and, in some cases, establish crosstalk (Tropberger et al., 2013; Wojcik et al., 2018; Kirsch et al., 2020), which introduce minor conformational alterations, allowing the recognition by PTM readers and initiation of the downstream activities (Taverna et al., 2007; Sanchez and Zhou, 2011). The dysregulation of PTMs can cause severe health issues such as cancers, neurodevelopmental disorders, and cardiovascular diseases (Schwartzentruber et al., 2012; Kim et al., 2017; Wickramasekara and Stessman, 2019; Zhao and Shilatifard, 2019; Bryant et al., 2020; Bagert et al., 2021). Furthermore, many nucleosome binding proteins recognize PTMs and cooperate with the modifications to accomplish biological functions, for example, H3K9me3 with HP1α, the PWWP domain with H3K36me3, and the SAGA complex with H3K4me3 (Vermeulen et al., 2010; Horn and van Ingen, 2020). Methylation is the most studied histone PTM at both molecular and genome levels. Structural studies showed that the dimethylation or trimethylation of H4K79 in NCPs result in subtle lysine sidechain structural rearrangements without global structural changes (Lu et al., 2008). It was recently revealed that the monomethylation of H4K20 leads to enhanced mobility of histones and less folded nucleosome arrays (Shoaib et al., 2021). This provides a molecular basis for the *in vivo* observation that H4K20me1 and H4K20me3 are accumulated at transcriptional active and suppression regions, respectively, which illustrate that the biological consequences of modifications are achieved through altering the dynamics of nucleosomes and, therefore, changing the compaction of nucleosome and the accessibility of DNA.

Acetylation is another prevalently occurring PTM that is crucial for DNA activities and reduces the net positive charge on histones. H4 tail acetylation likely leads to destabilizing chromatin at DNA double-strand breaks and dynamic changes of different modifications of the tail potentially regulate the repair pathways (Dhar et al., 2017). The genetically encoding acetyl-lysine strategy was used to provide large quantities of H3K56Ac, allowing a smFRET study that revealed the seven-fold increase in DNA breathing by this epigenetic modification (Neumann et al., 2009). An all-atom 5- to 6-microsecond MD simulation illustrated that acetylation of H3K56 weakens DNA–histone interactions and leads to further increase in mobility and exposure of DNA sites in lesion-containing nucleosomes, suggesting that this modification prepares the complex for DNA repair (Cai et al., 2020; Fu et al., 2021). In line with this, the combination of magnetic tweezers and FRET measurements showed that nucleosomes containing acetylation at the entry-exit site H3K56 or H4K77/K79 exhibited significantly enhanced DNA unwrapping (partial peeling of DNA ends from HO) and no change in disassembly (complete dissociation of DNA from HO) in comparison with unmodified NCPs (Simon et al., 2011). On the other hand, opposite effects were observed for nucleosomes harboring acetylation at the dyad site H3K115/K122 (Simon et al., 2011). Similarly, a FRET study of 170 bp Widom 601 nucleosomes revealed that acetylation of H3 and H4 induce different effects on nucleosome stability, where the former enhances DNA end unwrapping and the latter leads to opposite effects on disassembly and dimer exchange (Gansen et al., 2015). Those observations suggest that acetylation modifications occur at individual histone tail positions and independently modulate nucleosome dynamics through distinct mechanisms.

Besides acetylation, other lysine acylation modifications such as glutarylation and succinylation were also detected for histones *in vivo* (Li and Li, 2021). Glutarylation is a novel histone modification mark that was recently identified at 27 sites of histones (Tan et al., 2014; Bao et al., 2019). A study showed that glutarylation of H4K91 was highly enriched in active genes and the de-glutarylation was associated with chromatin condensation (Bao et al., 2019). FRET experiments revealed that glutarylation of H4K91 led to less stable nucleosomes in comparison with the acetylation of this site and the wild-type, and promoted the separation of H2A/H2B dimers from H3/H4 tetramers during nucleosome disassembly (Bao et al., 2019) Succinylation was first observed for all four histones by isotope labeling and HPLC/MS/MS analysis, and mutations on the succinylation sites led to functional consequences as demonstrated in budding yeast (Zhang et al., 2010; Xie et al., 2012). In comparison with acetylation, the succinylation introduces a longer sidechain and further reduction of the charge by one more unit due to the introduction of a negative carboxylate at the modified lysine site, therefore likely leading to greater alteration on structure and dynamics of the histones. The first site-specific succinylation-modified histones were obtained using thiol-ene addition at the H2BK34 site, and a smFRET study showed that the modification greatly attenuated DNA–histone interactions and reduced nucleosome structural stability (Jing et al., 2018). Succinylation of a nucleosome lateral surface residue, H3K122, leads to enhancing chromatin dynamics, which explains its transcription stimulation effects *in vitro* and enrichments in promoters of active genes *in vivo* (Zorro Shahidian et al., 2021).

Ubiquitination has been identified for tens of sites in histones and often establishes crosstalk with other modifications to regulate chromatin (Han et al., 2013; Mattiroli and Penengo, 2021). The unfolding of the outer DNA wrap in the nucleosomes harboring unmodified and ubiquitinated H2A required a free energy of 32 kJ/mol and 210 kJ/mol, respectively (Xiao et al., 2020). This ubiquitination achieves such effects through suppressing DNA unwrapping and, therefore, modulating the stability of nucleosomes. A study suggested that H2BK120Ub impairs the divalent cation-induced chromatin fiber compaction by affecting the later stage of compaction, while H4 acetylation disrupts the process *via* altering the electrostatic interactions at the early stage of compaction (Fierz et al., 2011). By combining a hydrogen–deuterium exchange strategy with NMR, it was revealed that H2BK120Ub results in decompaction of fibers likely mediated by the glutamate patch and ubiquitin fragments of neighboring mononucleosomes, interacting to hinder chromatin fiber association (Debelouchina et al., 2016). Phosphorylation increases the capability of forming electrostatic interactions with spatially closed chemical groups and contributes to DNA processes such as apoptosis, replication (Baker et al., 2010), stimulation-induced transcription (Armache et al., 2020), and telomere silencing (Zhang et al., 2021). The combination of adding negative charges and a bulkier side chain by phosphorylation of H3T118 resulted in a reduction of DNA–histone binding by 2 kcal/mol, an increase in DNA accessibility near the dyad by six folds, and the promotion of nucleosome disassembly by a remodeler (North et al., 2011).

The composition of DNA in nucleosomes is one of the dominant factors dictating the architecture, compactness, and accessibility of chromatin. Varying DNA sequences lead to changes in nucleosome structure, dynamics, positioning, and compactness (Shaytan et al., 2017; Shi et al., 2020b; Soman et al., 2020). For example, our recent study revealed that the telomeric NCPs exhibit higher mobility in both histone N-terminal tails and core regions in comparison with the Widom 601 NCPs (Shi et al., 2020b). Alteration of DNA flexibility by changing the sequence was found to modulate the unwrapping direction, where DNA unwraps more from the stiffer end, which can be facilitated by the stability of the inner turn of the DNA (Ngo et al., 2015). MD simulations of DNA minicircles yielded an energy landscape analysis showing that changing DNA sequence and methylation states induced conformational and energetic perturbations for the systems (Yoo et al., 2021). Experimental studies of structure and dynamics for DNA methylations have been lagging behind, partially due to the difficulty of large-scale methylated DNA preparation. A recently developed synthetic strategy utilized ^13^CH_3_-methionine, S-adenosylmethionine synthase, ATP, methyltransferase, and target DNA to produce ^13^CH_3_-methyl-labeled for solution-state NMR experiments. It successfully observed structure and dynamics information for DNA-methylated mononucleosomes (Abramov et al., 2020). The 5-hydroxymethylated cytosine (5 hmC) naturally occurs 10–100 times less than 5-methylcytosine (5 mC) and, different from 5 mC, it likely accumulates at euchromatin (Chen et al., 2014). The combination of FRET with a biochemical study observed that 5 hmC decreases nucleosome stability (Mendonca et al., 2014). These studies lead the way to understanding the mechanisms of chromatin activities modulated by post-translation modifications of DNA.

### Effector Proteins Altering the Dynamics of Nucleosome–Protein Complexes

DNA regulation is achieved through consecutive processes precisely cooperating at the temporal and spatial domain. For example, “writers” generate histone PTMs to open or tighten nucleosomes, which will be responded to by “readers” to incorporate regulatory proteins to interact with chromatin to trigger the downstream activities. The binding of effector proteins typically introduces essential changes to the structure, dynamics, and/or fiber compaction of chromatin, which often correlates with contacting interfaces. Yeast pioneer transcription factor Rap1 binds to chromatin fiber, resulting in no substantial structural disruption to the nucleosomes; instead, it interferes with the neighboring nucleosome interaction and opens chromatin (Mivelaz et al., 2020). Linker histone H1 is a key chromatin high-order structure modulating protein and contains the globular domain that binds to the nucleosome on the dyad (Bednar et al., 2017; Hao et al., 2021; Wang et al., 2021; Zhou et al., 2021), an N-terminal tail enhancing DNA binding (Collepardo-Guevara et al., 2020), and a C-terminal region interacting with linker DNA (Bednar et al., 2017; Hao et al., 2021; Wang et al., 2021; Zhou et al., 2021). The C-terminal domain retains high flexibility that allows H1 interacting with prothymosin *α* through highly disordered regions, promoting the dissociation of H1 from nucleosomes (Heidarsson et al., 2022). H1 undergoes structure changes upon binding to nucleosomes and alters the DNA accessibility by combining with PTMs and effector proteins (Collepardo-Guevara et al., 2020). H1 could bind to nucleosomes with on-dyad and off-dyad modes with the former more energetically favorable and the latter more dynamic (Wereszczynski and Woods, 2020; Rudnizky et al., 2021). The transition between the two modes may combine with other factors and serve as a switch for modulating DNA processes. PTMs spanning the entire protein are widely identified for H1 and many are revealed as linked to chromatin condensation/decondensation (Izzo and Schneider, 2016; Roque et al., 2016; Andrés et al., 2020). The acetylation of H1K85 likely results in a more condensed chromatin organization *via* enhancing its interaction with the histone core as demonstrated by using the modification mimic H1K85Q and also facilitates recruiting HP1 onto chromatin (Li et al., 2018). Phosphorylation modulates the structure of the H1 C-terminal domain and disrupts the condensation states of chromatin depending on the degree of modification (Roque et al., 2008; Izzo and Schneider, 2016). Comprehensive characterization of how H1 PTMs impact chromatin compaction and dynamics at the molecular level is generally lacking and awaits future investigation. The FACT complex is a histone chaperone that facilitates nucleosome assembly and disassembly, of which the mechanisms were recently revealed by cryo-EM structures of FACT–subnuclosome complexes (Liu et al., 2019). The binding of yeast FACT to a mononucleosome led to ATP-independent reversible DNA uncoiling involving >70% of the nucleosomal DNA as observed by FRET measurements for nucleosomes fluorescently labeled at three different sites (Valieva et al., 2016). A study combining solution-state NMR and FRET suggested that the human PHF1 Tudor domain binding to H3K36me3 containing NCP lead to the increase in nucleosome dynamics by shifting the population to the nucleosome opening state (Musselman et al., 2013). Cryo-EM combined with smFRET experiments showed that human methyltransferase DOT1L destabilizes nucleosome without alteration of HO conformation, and the effect is further enhanced by H2BK120 ubiquitination (Jang et al., 2019). In contrast to those effector proteins, chromatin-associated proteins such as HP1 contribute to the compaction of the chromatin fiber. Three isoforms, HP1α, HP1β, and HP1γ exist in mammalian cells. A recent cryo-EM study resolved 11.5–23.9 Å structures for the non-phosphorylated HP1 in complex with H3K9me3-containing dinucleosome, and revealed that HP1 forms a dimer that bridges two nucleosomes with linker DNA exposed to solvent (Machida et al., 2018). Another smFRET study elucidated that HP1α binds to nucleosomes on the 50–500 ms timescale and stabilizes chromatin fibers but introduces structural fluctuation on the sub-second timescale (Kilic et al., 2018). Taken together, the association of effector proteins with chromatin typically introduce changes to the dynamics and compaction of chromatin, preparing for downstream activities. There are often critical conformational changes occurring in many of those interactions, which are not fully characterized due to the limitation of techniques and await future studies.

### Histone Variants

Cells utilize the incorporation of histone variants to regulate gene events such as gene expression, DNA repair, and X chromosome inactivation (Sarma and Reinberg, 2005; Biterge and Schneider, 2014; Martire and Banaszynski, 2020). The histone variants, H3.2, H3.3 and CENP-A, H2AZ, H2AZ, and microH2A, share similarities of 50–99% with canonical ones and introduce unique compaction and accessibility features to chromatin (Sarma and Reinberg, 2005; Biterge and Schneider, 2014; Nechemia-Arbely et al., 2017). CENP-A is found at the active centromeres and its misregulation is observed in cancers. In comparison with the canonical NCP, the human CENP-A–containing NCP possesses a structure with thirteen base pairs at both ends of DNA absent and CENP-A αN loop shortened, suggesting increased flexibility of those regions (Tachiwana et al., 2011). As elucidated by FRET, the replacement of H3 by CENP-A leads to a destabilized and reshaped nucleosome structure and requires the binding of CENP-C to stabilize to a similar shape to that of the canonical nucleosomes (Falk et al., 2015; Falk et al., 2016). H2AZ2.2, a histone H2AZ variant, is demonstrated to be existing *in vivo*, and it functions by destabilizing nucleosomes, mainly attributed to its C-terminal region weakening the interactions with H3 (Bönisch et al., 2012).

## Conclusion

Our understanding of the atomistic details of structure and dynamics of nucleosomes and nucleosome–protein complexes has been significantly expanded with the last two decades’ development of high-resolution techniques. Here, we summarized studies and their importance pertaining to the dynamics of nucleosomes and their changes induced by the presence of modulation factors including PTMs, histone variants, and effector proteins. The functional relevant motions in chromatin typically span from the microsecond to the sub-second window, and the dynamics alterations introduced by modulation factors are achieved by the cooperation of multiple dynamical regions. Due to technical limitations, particularly FRET, much of the currently elucidated dynamics information is still limited by spatiotemporal resolution; however, it indubitably illustrates that dynamics play dominant roles in chromatin regulation processes. In addition, because subtle conformational changes are hard to capture in many of those studies discussed here, we cannot exclude the significance of structure contribution in this context. Ideally, combining atomic structure and dynamics characterization in the future will allow the complete understanding of chromatin regulation mechanisms at the molecular level.
